# The Diaper Change Play: Validation of a New Observational Assessment Tool for Early Triadic Family Interactions in the First Month Postpartum

**DOI:** 10.3389/fpsyg.2018.00497

**Published:** 2018-04-13

**Authors:** Jérôme Rime, Hervé Tissot, Nicolas Favez, Michael Watson, Werner Stadlmayr

**Affiliations:** ^1^Faculty of Psychology and Educational Sciences, University of Geneva, Geneva, Switzerland; ^2^Center for Family Studies, University Institute of Psychotherapy, Lausanne University Hospital, University of Lausanne, Lausanne, Switzerland; ^3^Department of Child and Adolescent Psychiatry and Psychotherapy, Psychiatric Services Aargau AG, Aarau, Switzerland; ^4^Geburt und Familie – Praxis Dr. med. Werner Stadlmayr, Aarau, Switzerland

**Keywords:** early triadic interactions, family interaction, newborn, assessment, observational method, validity

## Abstract

The quality of family relations, observed during mother–father–infant triadic interactions, has been shown to be an important contributor to child social and affective development, beyond the quality of dyadic mother–child, father–child, and marital relationships. Triadic interactions have been well described in families with 3 month olds and older children using the Lausanne Trilogue Play (LTP). Little is known about the development of mother–father–baby interactions in the very 1st weeks postpartum, mostly because no specific observational setting or particular instrument had been designed to cover this age yet. To fill this gap, we adapted the LTP to create a new observational setting, namely the Diaper Change Play (DCP). Interactions are assessed using the Family Alliance Assessment Scales for DCP (FAAS-DCP). We present the validation of the DCP and its coding system, the FAAS-DCP. The three validation studies presented here (44 mother–father–child–triads) involve a sample of parents with 3-week-old infants recruited in two maternity wards (*n* = 32 and *n* = 12) in Switzerland. Infants from both sites were all healthy according to their APGAR scores, weight at birth, and scores on the NICU Network Neurobehavioral Scale (NNNS), which was additionally conducted on the twelve infants recruited in one of the maternity ward. Results showed that the “FAAS – DCP” coding system has good psychometric properties, with a good internal consistency and a satisfying reliability among the three independent raters. Finally, the “FAAS-DCP” scores on the interactive dimensions are comparable to the similar dimensions in the FAAS-LTP. The results showed that there is no statistically significant difference on scores between the “FAAS-DCP” and the “FAAS,” which is consistent with previous studies underlying stability in triadic interaction patterns from pregnancy to 18 months. These first results indicated that the DCP is a promising observational setting, able to assess the development of the early family triadic functioning. The DCP and the FAAS-DCP offer to both clinicians and researchers a way to improve the understanding of the establishment of early family functioning as well as to study the young infant’s triangular capacity. Perspectives for future research will be discussed.

## Introduction

Research about family functioning through the transition to parenthood has grown into an important area of inquiry. Many different instruments have been created and validated to assess family—or family-to-be—functioning in the peripartum period through the observation of family interactions. Prenatal instruments, such as the observational situation “Prenatal Lausanne Trilogue Play” (Prenatal LTP; Corboz-Warnery and Fivaz-Depeursinge, 2001; Carneiro et al., 2006; Favez et al., 2012b; Altenburger et al., 2014) can be used to foreshadow the future family functioning, whereas paradigms of observation of mother–father–child interactions, such as the “LTP” paradigm (Fivaz-Depeursinge and Corboz-Warnery, 2001; McHale et al., 2008; Tissot et al., 2015), have been designed to be used with families with infants from 2 or 3 months postpartum. However, no existing tools were specifically designed to assess triadic interactions in the very 1st weeks postpartum, which would allow the earliest detection of potential family distress. We have addressed this gap by developing a new observational assessment tool specifically designed to assess mother–father–newborn interactions in the 1st month postpartum. Here we present the validation of the Diaper Change Play (DCP) and its coding system, the Family Alliance Assessment Scales for DCP (FAAS-DCP).

### The Necessity of a Very Early Detection of Distressed Families

Various authors in past decades have highlighted the importance of studying family functioning through the observation of triadic interactions. The study of family functioning at a triadic level allows us to examine the three family subsystems, consisting of the parental subsystem (i.e., mother–child and father–child relationship), the marital subsystem (i.e., the relationship between partners/spouses), and the coparental subsystem (i.e., the relationship between the parents regarding their child). According to family systems theory, the family subsystems and the individuals within are interrelated, each of them contributing to the establishment of family relationships (Pinel-Jacquemin and Zaouche-Gaudron, 2009; Stroud et al., 2011). Moreover, it has been shown that the triadic family environment has a unique, specific, and independent influence on the child’s socioemotional development. For example, empirical evidences have shown that coparenting mediates the relation between marital and parental relationships (Bonds and Gondoli, 2007; Pedro et al., 2012), and that a healthy coparenting functioning is associated with the positive affective and social development of the child (Teubert and Pinquart, 2010). Conversely, coparenting characterized by a low level of support and a high level of conflict is associated with negative outcomes for the child. A child growing up in such an environment will be at risk, by preschool age, for developing behavioral problems such as aggression (McHale et al., 2008), and presenting depressive and anxious symptoms (Katz and Low, 2004).

The childbirth implies numerous reorganizations within the family system. Indeed, partners become parents as well as coparents. Parents need to adapt to the changes related to the child’s arrival and to these new roles. Thus, the immediate postpartum period is a critical and stressful moment for the new parents. Indeed, parents may experience problems in their marital relationship, in their parental and/or coparental roles and it is crucial to help them at soon as possible. The major risk for families who experience a significant distress in the very early days or weeks after childbirth is the emergence of dysfunctional relational patterns that may, in the long run, crystallize into problematic relationships among family members, which may in turn put the child at risk for developing socioemotional difficulties and psychopathological disorders by school age and during adolescence (Kelly, 2000; Cummings et al., 2012; Brock and Kochanska, 2016). Therefore, it is crucial to develop instruments that will allow detecting problematic relational patterns in the very early postpartum period.

In the context of the maternity ward, families have contact with many professional caregivers who are at the forefront to detect early relational difficulties in the families (Alder et al., 2006). Indeed, parents who feel helpless or overwhelmed by the stress associated with childbirth and parenthood may talk with midwives, obstetricians, nurses or psychologists. These caregivers may provide considerable help in the process of transition to parenthood (Ayers et al., 2007), as this period of intensive relations between families and health care professionals is a unique moment to detect early relational difficulties in the families. Moreover, it will be all the more crucial to detect these problems as early as possible, because later in the postpartum, parents—and especially mothers—who experience difficulties to establish positive relationships with their child may feel guilty or ashamed to seek for help. Indeed, they may be afraid to be considered as a “bad parent” (Edhborg, 2004; Riecher-Rössler and Rohde, 2005) or to be perceived by others as ineffective. Because health care professionals will be less in contact with families beyond the perinatal period and then less able to detect relationship problems, creating instruments to this end is thus especially important.

### The Contribution of the Observational Method

An observational tool to assess family interactions is chosen with the understanding that it provides privileged access to family functioning. The observation of interactive behaviors during family interactions allows understanding a set of rich, nuanced, and distinct interpersonal dynamics that are characteristic of family-level functioning (McHale et al., 1996), which could not be captured by individual measurement, like questionnaires or interviews (McHale et al., 2000). Indeed, whereas self-reported measures give an access to individual perceptions of the family relationships, the observational method permits investigation of aspects and dimensions of behaviors, beyond the awareness of the family. Moreover, it has been shown that what occurs in family-level relationships, for example during mother–father–infant triadic interactions, are distinct from dyadic mother–child and father–child relationships and could not be inferred by the observation of separate dyadic interactions (Kerig, 2001; Lindahl, 2001). Indeed, given that each parent will behave differently if the other parent is present or absent, observing triadic interactions permits consideration of the active role of each simultaneously and highlights their individual contributions (von Wyl et al., 2008; Venturelli et al., 2015; Udry-Jørgensen et al., 2016). Finally, the observation of the triad allows to include the infant and to take his influence on the family system into account (Rime and Stadlmayr, 2013).

Given the lack of observational tools to assess the triadic interactions between the prenatal period and the 3 months postpartum, we created the DCP to allow a specific assessment of triadic interactions in the early postpartum. As a theoretical background, we referred to the family alliance model, as well as the empirical results of the studies on later triadic family functioning using the LTP.

### Conceptualisation of Family Functioning Through the Concept of Family Alliance

To guide the evaluation of family functioning, we based our approach on the model of family alliance developed for the LTP (Fivaz-Depeursinge and Corboz-Warnery, 2001). This model considers the degree of coordination and engagement in a shared activity as an index of the quality of family-level relationships. The terms “family alliance” corresponds to the capacity of the family to coordinate and create a favorable context for a mother–father–infant communication (Fivaz-Depeursinge and Favez, 2006). Specifically, the parents provide both predictable and adapted stimulations in response to the state and skills of infant. The infant addresses signals to parents about his state, which permit them to adjust their behavior. The parents and the infant have reciprocal and mutual influences, but the parents occupy a superior hierarchical position, in comparison of the infant, that allows them to provide a favorable context for the child’s development.

To assess the quality of family alliance, the characteristics of its structural and dynamic foundations should be considered (Favez et al., 2011; Frascarolo et al., 2004). The first foundation refers to four interlocked communication functions for attaining harmonious interactions. Achievement of each function is a prerequisite for the next function. The first function is called “participation” and refers to the requirement that all family members should be included; that is, they should demonstrate availability and readiness to interact. In concrete terms, availability is signaled by body orientation and positioning that creates a suitable space to interact. The second function is “organization,” which requires all members to respect their role in the interaction. For example, if the two parents are together to share a moment with the infant, they should have an equivalent opportunity to interact (neither one nor the other should be excluded from the interaction). Basically, the role of parents is to provide adjusted and predictable stimulations and the child’s role is to send signals to enable parents to adjust to his state. When all members are included and maintaining their roles, they should focus their attention on the same subject to enable participation in a shared activity. This third function, characterized by the orientation of the gazes and the sharing of a common activity is called “focalization.” The last function, called “affect sharing,” refers to the circulation of affects between family members, and involves sharing genuine emotions and displaying emotional interest to each other. Empathy and affects validation and sharing are the clues for confirming achievement of this last communicative function. Together, these four interactive functions describe the structure of the interactions.

The second foundation relates to the dynamics or temporal aspect of the interactions. Each interaction is marked by fluctuations, pauses in activities, and changes in the content of the exchange. These variations in the interactions involve transitions and adjustments. However, interactive mistakes, like a lack of synchrony or interruptions are unavoidable in the flow of natural interactions (Tronick and Cohn, 1989; Fivaz-Depeursinge and Corboz-Warnery, 2001). Thus, the family alliance is also defined by the ability to deal with these interactive mistakes, fluctuations, pauses, and changes in a way that keeps interactions as flowing and consistent as possible (Frascarolo et al., 2004). The dynamic/temporal dimension is transversal to the four interactive functions.

When taking the two foundations into account, it is possible to consider several different kinds of alliances, namely cooperative, collusive, and disordered. The cooperative alliance is characterized by an inclusion of all family members, the respect of the roles of each member, and congruent exchanges and affects sharing that is more or less successful according to the ease and emotional regulation ability of each family member^1^. The collusive alliance refers to a situation where all family members are included, but their roles are not respected. A competition between parents is observed and the rhythm of the exchanges is too fast, leading to an over-stimulation of the infant. The interactions are marked by interferences. A covert or overt conflict causes a split in the coparental unity. The disordered alliance is representative of families where the participation of all members is not possible. One of the members is excluded (or excludes himself) from the interaction. The exchanges are marked by attitudes of indifference, withdrawal, and a lack of connection either between the parents or between the parents and the baby. Exchanges start but stop abruptly. The cooperative alliance facilitates the understanding of the diverse three-ways relational configurations (Frascarolo et al., 2007a). The infant receives consistent and appropriate stimulations that facilitate the comprehension of the situation involving the parental roles and the resulting communication behaviors. This allows the infant to develop triangular processes that, in turn, allows the establishment and maintenance of the triadic relationships. Collusive and disordered alliances are considered, respectively, as problematic and dysfunctional. The poorer the quality of the family alliance is, the more it compromises the establishment of triadic relationships due to a predominance of inherent confusion and negativity.

As our aim was to develop a specific observational situation designed to assess family alliance in the 1st month postpartum, we created a setting that will be representative of the daily routines of the family. Whereas the LTP is based on a play task, the DCP is mainly based on a caregiving task. Indeed, the interactions are observed during the change of diapers. The choice of the diaper change activity is relevant for several reasons. First, this activity is a daily routine in which intuitive parenting behaviors are involved. Just like others daily routines, it allows the newborn to establish a reciprocal relationship with his parents. Moreover, diapering presents an excellent way to observe interactions and represent a crucial activity in which the newborn can experience affective exchanges, affect sharing and then developing socioemotional skills (Montague and Walker-Andrews, 2002; Addessi, 2009). Secondly, observing this care activity, even in a lab-based setting, allows data to be collected with increased ecological validity (Bornstein et al., 2011). The diapering activity provides a window into the reality of family functioning and then the quality of exchanges between partners viewed through one major activity in early postpartum. Finally, working on the diaper change could be an interesting way to help parents who feel helpless in their parental role to develop a higher sense of parental self-efficacy, and help to mitigate negative consequences for the parents-infant relationships (Papousek, 1996; Papousek and Von Hofacker, 1998).

In the present paper, we present the first steps toward the validation of the DCP and its coding system, the FAAS-DCP. This new observational situation and its coding system have been, respectively, inspired by the LTP and its coding system, the Family Alliance Assessment Scales (FAAS). To that end, we present three studies: The first study aims to establish the inter-rater reliability in order to verify if different coders have the same understanding on the manner to rate the interactions. Then, the second study aims to establish the reliability and the internal consistency in order to verify if our interactive dimensions are consistent with the theoretical construct of family alliance. The third study aims to establish the predictive validity in verifying if the evaluation of the family functioning observed at 3 weeks during the DCP is similar of the one observed at 3 months during the LTP.

## Method

### Population

The three validation studies involve one sample drown from two maternity wards, one in Breitenbach and the other in Bern, Switzerland. All of the families in the sample were Caucasian European families.

This sample consists of 44 volunteer parents and their newborns. 32 families coming from Breitenbach’s maternity ward participated in a longitudinal study from pregnancy to the end of the 1st month postpartum (FNS grant no. 3200-049741.96). 12 families coming from the maternity ward at Bern have participated in a longitudinal study from pregnancy to the 1st year postpartum (FNS grant no. 105314-127121). The studies had been approved by local ethical committees [sample Breitenbach: Ethikkommission Beider Basel EKB (250/00); Sample Bern: Kantonale Ethikkommission Bern KEK (110/07)]. The decision to bring families from the two studies together to form our sample is based on the need to increase statistical power. It was useful to combine data collected from the two maternity wards to consolidate analyses on the psychometric properties of the coding system FAAS-DCP. Mother’s mean age was 31.4 (*SD* = 4.36) at the birth of the child. Father’s mean age was not calculated, since this information was missing from the data collected for the Breitenbach maternity ward.

Firstborn children constituted 40.9% of the sample. No statistical difference was found between primiparous and multiparous women on each interactives dimensions (Z score ranged between 0.265 and 1.073; *p* > 0.05). Likewise, no difference was found between those two groups of women on the family alliance category (χ^2^(1) = 0; *p* > 0.05). Therefore, primiparous experienced a similar stress level compared to multiparous, so that it did not influence triadic family functioning. 25 newborns were boys and 19 were girls. In five cases (11.4%), the newborn was born prematurely (at less than 37 weeks of gestation). All newborns had an APGAR score of 9 or 10, meaning they were all in good health. Newborns from Bern’s maternity ward were also examined with the NICU Network Neurobehavioral Scale (NNNS), providing an examination of neurologic integrity and behavioral functioning (Lester and Tronick, 2004; Liu et al., 2010). Specifically, motor development, muscular tonus, neurological reflexes and behavioral organization were assessed to inform an opinion about the newborn’s health. This examination highlighted that all newborns showed good visual and auditory attentional capacities. They showed satisfactory capacities to sustain attention too. They were easily soothed and showed no signs of neurological injury or behavioral problems. All newborns were in good health when they were recruited for our study.

### Procedure

Families from Breitenbach came to their local maternity ward where family interactions were video-recorded during the DCP at 3 weeks postpartum. Families from Bern came to their local maternity ward where family interactions were also video-recorded during the DCP at 3 weeks postpartum, and then again at 3 months postpartum during the LTP (Corboz-Warnery et al., 1993). All families gave their written consent.

#### The Diaper Change Play

The DCP is a situation involving the mother, the father and the newborn. It was initially developed by Dr. Stadlmayr on the basis of the LTP structure, and adapted to assess early triadic interactions with a 3 weeks’ postpartum newborn. The idea of play was changed to a practical activity approximating the daily life, involving a caring activity. In the first version, used with the Breitenbach sample, the parents sit in chairs with armrests positioned in front of and on each side of the infant, who is lying on a changing mat on a rectangular table fixed to a rotating axle. The bodies of the parents and the infant are positioned to form a triangle. The technical equipment includes two cameras; one records the parents, the other the newborn.

**FIGURE 1:**
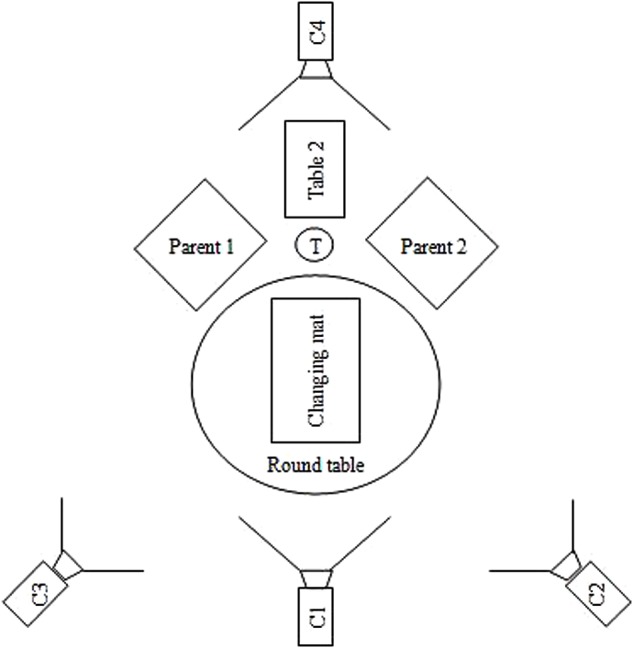
Aerial view of final DCP setting. Round Table: table where rests the changing mat; Changing mat: fixed on the round table; **Table 2**: table on which the material to change the diaper is disposed; T: trash; C1: Camera 1: allow a general view of the two parents; C2, C3: Camera 2 and 3: allow to have a close-up on the two parents’ face; C4: Camera 4: allow to have a close-up of the infant.

**FIGURE 2:**
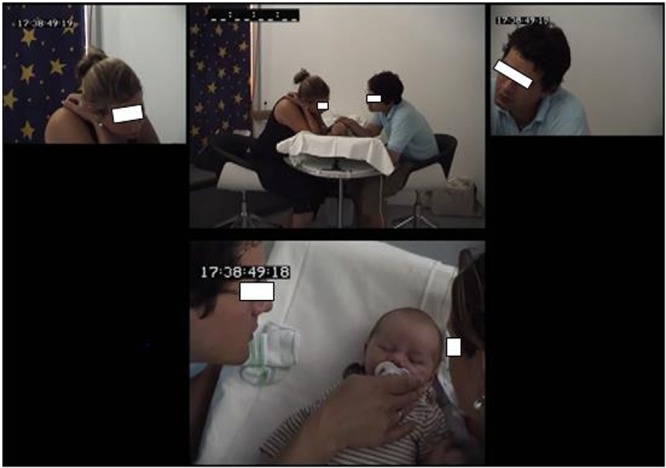
Video editing from the four cameras.

This initial setting was modified for the families in the Bern sample after some limitations were observed with the Breitenbach sample. The changing table was inclined 10–15 degrees to provide a better angle for interaction between the infant and parents (von Hofsten, 1982; Rochat, 1992). The rectangular table was replaced with a round table, and pivoting chairs without armrests replaced the original chairs. These changes improved the triangular configuration and facilitated movement on the part of the parents during the activity (see the **Figure 1** for a rendering of the setting). The technical equipment was also changed to provide four cameras in order to record the newborn, the parents together, and each parent separately. Images from the cameras were synchronized together with Adobe Premiere Element software^®^ to allow an assessment of the interactions viewed from these different sources (see **Figure 2** for the result of video editing).

The admistration of the DCP procedure was similar in both samples. The DCP is structured in four parts. In the first part, after the parents agree on who starts the activity, one parent begins to change the diaper of the baby and the other parent observes the interaction without intervening. The roles change in the second part, when the parent previously in the observer role becomes active and finishes the process of changing the diaper. Meanwhile, the previously active parent attentively observes the interaction. In the third part, the parents are together with the baby to share a moment. The parents might stroke the baby, smile at him, or soothe him if required. The fourth part is the marital moment during which we asked the parents to have a discussion in the presence of the baby. Parents should be oriented toward each other, to share this marital moment, while remaining attentive to the baby’s state.

#### The Family Alliance Assessment Scale for DCP (FAAS-DCP)

As said before, the coding system “FAAS-DCP” is modeled on the FAAS (Lavanchy et al., 2006). A first version was built on the framework of a Master’s project (Delévaux et al., 2006). On this basis, a working group was set up with the help of the “Mutterhilfe” foundation. The team was comprised of five experimented clinicians (family therapists and researchers) involved in experimental studies, including Dr. Stadlmayr who created the observational assessment tool DCP. Different versions of the FAAS-DCP were tested and considered; and the final version, discussed here, represents the consensus of the working group.

The FAAS-DCP consists of 9 interactive dimensions, respectively, named “readiness to interact,” “gaze orientation,” “inclusion of partners,” “coparental coordination,” role organization,” parental scaffolding,” “shared and co-constructed activities,” “sensitivity,” and “family warmth” (see descriptions in **Table 1**).

**Table 1:** Description of the 9 interactive dimensions of the FAAS-DCP.

Interactive dimensions	Description of the assessment
1. Readiness to interact	It assesses the physical signs, facial expressions, and attitudes signaling an availability to interact. The newborn’s availability to interact is based on the identification of newborn’s states as defined for the NNNS examination (Lester et al., 2004). Briefly, state 1 refers to an newborn who sleeps soundly; state 2 refers to an newborn who sleeps less soundly and exhibits some motor activity; state 3 refers to a drowsy newborn; state 4 refers to an awake and alert newborn; state 5 refers to an newborn who presents a considerable motor activity, potentially with brief fussy vocalizations; and state 6 refers to a newborn who is crying.
2. Gaze orientation	It assesses if gazes are is oriented within the triangular space of the interaction and if an eye contact is established with the newborn. This space is defined by the body positions of the mother, the father and the newborn.
3. Inclusion of partners	It assesses the inclusion of each family member. Behaviors constituting inclusion, self-exclusion (for example, when one parent withdraws from the interaction when he is supposed to participate), and hetero-exclusion (for example, one parent ignore the other) are evaluated.
4. Coparental coordination	It assesses cooperation and support between the parents, as well as intrusive or interfering behaviors occurring between them, and their overall coordination with each other during the activity. The presence of supportive behaviors directed at each other and overt or covert conflict are evaluated.
5. Role organization	It assesses the way each partner respects and expresses his assigned role in the activity and how the partners jointly negotiate the organization of the activity. The infant’s role is to send signals to his parents (movements, vocalizations, crying, etc.).
6. Parental scaffolding	It assesses the quality of parental stimulations and care, which should be predictable and adjusted to the newborn’s state and skills.
7. Shared and co-constructed activities	It assesses the ability of the parents to co-construct an activity taking into account of the newborn’s state. The shared interactions, including discussions, involved in carrying out the care activity are assessed. Co-construction is reached when each partner contributes to the evolution of the exchange.
8. Sensitivity	It assesses the validation and regulation of the newborn’s affect by the parents and if they respond empathically to their infant’s emotions. Sensitivity is defined by Ainsworth et al. (1978) as the ability to correctly “read” affects; it allows the validation of the newborn’s affects.
9. Family warmth	It assesses the affective tone of the interactions. Specifically, it evaluates how well positive or negative affects circulate between family members and if they are genuinely shared. It concerns the richness and harmony of the family’s emotional atmosphere.

These 9 interactive dimensions are rated on a 5 points scale with a score of 5 representing the optimal functioning, and score of 1 representing significant dysfunction. The scores are assigned according to specific criteria detailed in the coding system FAAS-DCP. Given that the observational situation DCP is divided in four parts, a score is attributed to each of the parts. Then, the score on each dimension is obtained by summing the score on each part, ranging from 4 to 20. The second part of the evaluation involves a categorical assessment of the quality of family alliance in terms of cooperative, collusive and disordered alliance (see **Table 2**).

**Table 2:** Description of the family alliance categories according to the communicative function and the dynamics of interactions.

Categories of family alliance	Description of criteria
Cooperative alliance	All family members are included in the interactions; the role of each member is respected and a common activity is shared; affect sharing is more or less successful according to the ease and the capacity of emotional regulation of family members.
Collusive alliance	All family members are included in the interactions; organization function is not fulfilled because the role are not respected and a competition is visible between parents; the rhythm of the exchanges is too high and provoke an over-stimulation of the newborn.
Disordered alliance	Participation function is not fulfilled and one (or several) other family member is excluded (or excludes himself).

#### Other Observational Tool: The Lausanne Trilogue Play (LTP)

The LTP is a semi-standardized play situation involving the parents and the infant together. The infant is placed in a chair specially designed to accommodate the child’s weight and size. The parents sit in front of and on each side of the child. The child is oriented either between the two parents or oriented toward one of them due to the adjustable chair. As in DCP, the bodies of the parents and the child are positioned to form a triangle. The technical equipment includes four cameras: one camera records parents together, two cameras record each parent separately and one records the infant. In the LTP, parents are asked to play with the baby. The play is structured in four parts, corresponding to the four possible relational configurations in a triadic setting. In the first part, one parent plays with the baby and the other parent attentively observes the interaction. In the second part, the parents reverse roles and the active parent becomes an observer, while the previously observing parent becomes active. In the third part, the parents and the baby play together. In the last part, the parents converse together while the baby is in the third party position.

The FAAS coding system includes the same interactive dimensions and conceptualisation of family alliance as those of the FAAS-DCP. However, this coding system has one more dimension that rates the active participation of the baby. As this dimension is not relevant in the context of FAAS-DCP, we decided to exclude it from the analyses comparing the two coding system. The establishment of interactives scores work in the same way as the FAAS-DCP. For an exhaustive presentation of the FAAS, we refer to an unpublished work of Favez et al. (2006b).

### Coding Strategy

All of the videos recorded in the DCP and LTP were coded by one coder. Two additional coders were formed to the evaluation of triadic interactions in order to establish the inter-rater reliability. They coded separately the same videos. 21 video tapes were randomly selected from the Breitenbach maternity ward. All the coders were not aware of the status of the families. We used the intra-class correlations (ICC) to establish the inter-rater reliability [see section “Study 1: Inter-Rater Reliability (*n* = 21)].

## Results

### Study 1: Inter-Rater Reliability (*n* = 21)

This first study aimed to verify that the use of the coding system FAAS-DCP yields similar results from different raters. In other words, we have sought to establish the degree of consensus between different clinicians and researchers in the evaluation of family functioning on the basis of observed interactions in the observational situation DCP. To estimate the degree of consensus, we have used the threshold established by Ebel (1951) who defined the intra-class correlation (ICC) as follow: ICC < 0.4 = weak consensus; ICC ranging from 0.04 to 0.75 = moderate consensus; ICC ≥ 0.75 = strong consensus.

The inter-rater reliability (ICC) was ranged from 0.66 to 0.82 and all correlations were significant (*p* < 0.05). The interactive dimensions “readiness to interact” (ICC = 0.82), “gaze orientation” (ICC = 0.75), “inclusion of partners” (ICC = 0.82), “parental scaffolding” (ICC = 0.82), “sensitivity” (ICC = 0.77) and “family warmth” (ICC = 0.75) were the most reliable. Since raters showed a strong consensus, that means a shared understanding of theses aspects of family functioning were observed in the triadic interactions. The raters showed a satisfactory degree of agreement for the dimensions “coparental coordination” (ICC = 0.67), “role organization” (ICC = 0.74), “shared and co-constructed activities” (ICC = 0.66). We could conclude that the coding system FAAS-DCP is suitable to assess triadic interactions on the basis of the 9 interactive dimensions.

The degree of agreement was also calculated for the establishment of family alliance. We highlighted a strong consensus between the three raters (ICC = 0.76). Therefore, the descriptions of the different family alliance categories were well defined and permitted a shared understanding. These categories could be used to consider the triadic family functioning.

### Study 2: Reliability and Internal Consistency of the Coding System FAAS-DCP (*n* = 44)

This second study aimed to verify the homogeneity of the interactive dimensions of the coding system FAAS-DCP. In other words, we are looking at whether each dimension contributes to the assessment of the family functioning.

Scores on each dimension were consistent with the family functioning qualified by the family alliance (see **Table 3**). Indeed, families that had showed a more appropriate triadic functioning obtained higher scores on interactive dimensions. Nonetheless, mean scores increased on “coparental coordination” and “role organization”, respectively, for collusive and disordered family alliances.

**Table 3:** Mean scores, standard deviations, and range on the 9 interactive dimensions.

		Readiness to interact	Gaze orientation	Inclusion of partners	Coparental coordination	Role organization	Parental scaffolding	Shared activities	Sensitivity	Family warmth
**Harmonious alliances (*n* = 22)**										
	*M*	16.27	14.14	17.14	15.27	15.45	17.18	17.09	18.18	17.00
	*SD*	1.58	2.34	1.70	2.16	2.61	2.22	2.22	1.79	2.09
	Range	12–19	9–18	13–20	11–19	10–20	13–20	11–20	14–20	12–20
**Collusive alliances (*n* = 12)**										
	*M*	14.50	12.58	13.08	10.92	11.50	16.25	13.83	16.67	12.08
	*SD*	3.26	2.39	2.50	1.08	2.07	2.05	1.70	2.39	2.50
	Range	8–18	9–17	9–17	9–12	9–16	13–20	12–17	12–19	8–18
**Disordered alliances (*n* = 10)**										
	*M*	11.00	11.10	10.60	12.60	12.50	14.00	10.60	13.70	10.50
	*SD*	2.11	2.56	2.37	1.96	2.46	2.11	1.96	1.89	1.51
	Range	7–14	8–16	8–15	10–16	8–16	11–17	8–14	10–16	8–13
**Total (*n* = 44)**										
	*M*	14.59	13.02	14.55	13.48	13.70	16.20	14.73	16.75	14.18
	*SD*	3.05	2.65	3.44	2.65	2.99	2.45	3.32	2.64	3.56
	Range	3–19	5–19	6–20	5–19	5–20	7–20	2–20	6–20	4–20

Then, we conducted a discriminant analysis to determine if the 9 interactive scores allow us to determine the quality of the family alliance. The interactive dimensions were therefore considered as predictors of attribution of the three family alliance categories (i.e., harmonious, collusive, or disordered). The Kolmogorov–Smirnov test of normality highlighted that scores at the interactive dimensions were not normally distributed. The dimension “role organization” was the only one to be normally distributed within each family alliance category [*D*(22) = 0.177, *p* > 0.05; *D*(12) = 0.179, *p* > 0.05; *D*(22) = 0.181, *p* > 0.05]. The multivariate normality was therefore not respected. Moreover, variances were not homogeneous between groups for the dimensions “readiness to interact” [*F*(2.41) = 5.255, *p* < 0.05] and “coparental coordination” [*F*(2.41) = 3.855, *p* < 0.05]. The Box test showed also that homogeneity was not respected (*p* < 0.05). As suggested by Brace et al. (2006), despite the lack of homogeneity, it is possible to conduct a discriminant analysis if the analysis could highlight an accurate classification. Therefore, a test of equality of group means was performed and showed that the means score on each dimension differed significantly across family alliance (*p* < 0.05 for each dimension). The Wilks’ Lambda indicated that the dimensions “family warmth” (λ = 0.33), “inclusion of partners” (λ = 0.35) and “shared and co-constructed activities” (λ = 0.36) were those that best discriminate the different categories of family alliance. On the other hand, the dimensions “gaze orientation” (λ = 0.78) and “parental scaffolding” (λ = 0.73) were the less discriminant.

The discriminant analysis highlighted the existence of two discriminant functions. The value of the first function significantly differed according to family alliance categories (χ^2^ = 82, df = 18, *p* < 0.05). The second function showed characteristics similar to the first (χ^2^ = 21.02, df = 8, *p* < 0.05). Therefore, the discrimination between the alliance categories was based on two implicit factors. The interactive dimensions that were the most correlated to the first function were “family warmth” (*r* = 0.69), “inclusion of partners” (*r* = 0.66), and “shared and co-constructed activities” (*r* = 0.64). This analysis was partially consistent with the concept of communicative functions necessary to a triadic communication. Since affects circulation and shared activities are aspects of the “affect sharing” function, then families that present conflicts not fulfill this function. Therefore, these two dimensions contributed to the ability to discriminate between families with a collusive alliance and those with a cooperative alliance. However, it was surprising to note that “inclusion of partners,” that is related to the “participation” function and not fulfilled in disordered families, did not provide for better discrimination between collusive and disordered alliances (*r* = 0.07). An explanation is that collusive families often exhibited interferences that, if they were numerous and pronounced, could generate exclusions. However, families characterized by collusive or disordered alliances showed scores that were not sufficiently distinct to allow discrimination. It should be noted that the validation study for the FAAS underlined the need of a better specification of behaviors taking into account for an assessment on the dimension “inclusion of partners” (Favez et al., 2011). Given that the FAAS-DCP version of this dimension is based on that one for LTP, it is plausible that our criterion needed a similar specification. Nonetheless, it allows for discrimination with cooperative alliances that is consistent with the theoretical construct.

Moderate correlations were obtained on the first and second discriminant function, respectively, *r* = 0.44 and *r* = 0.38 for the dimension “readiness to interact” and *r* = 0.44 and *r* = 0.38 for the dimension “sensitivity.” It is consistent with the facts that these dimensions refer to the functions “participation” and “affect sharing” and also help to distinguish disordered and collusive families that are characterized, respectively, by a lack of participation and a lack of empathy [related to the conflict between parents (Frascarolo et al., 2007a)] when compared to cooperative families.

Likewise, the “parental scaffolding” dimension contributed to discriminate collusive from cooperative alliances as much as disordered from collusive alliances, but more modestly. Finally, the dimension “orientation gaze” was the less discriminant dimension, with correlations of *r* = 0.22 and *r* = 0.09, respectively, to the first and second discriminant function. Concerning the second discriminant function, the most relevant interactive dimension to discriminate collusive from disordered family alliances were “coparental coordination” (*r* = -0.67) and “role organization” (*r* = -0.40) that refer to “participation” and “organization” functions. Despite these negative correlations that mean the families with disordered alliances obtained better scores than families with collusive alliances, this result is consistent with the facts that participation and organization are the two unfulfilled functions within these families. Families with collusive alliances struggle to coordinate, and consequently do not fulfill the “participation” function. Families with disordered alliances are marked by exclusion and a lack of cohesion which prevents them from fulfilling the “participation” function. In summary, these interactive dimensions enable the discrimination between functional alliances and those that are problematic and dysfunctional.

The **Table 4** indicates a degree of accuracy of 88.6% for correctly predicting the family alliance category on the basis of the two discriminant functions. Specifically, the cooperative alliance was predicted best, with an accuracy of 95.5%. The least correctly classified alliance was the disordered alliance, with an accuracy of 80%. In cases where it was misclassified, it was identified as a collusive alliance. Finally, the collusive alliance was correctly classified in 83.3% of cases, with an equivalent risk of being misclassified as cooperative (8.3% of cases) or disordered (8.3% of cases). We can conclude that the discriminant functions are sufficiently accurate to enable a reliable discrimination between families with functional alliances versus those with collusive or disordered alliances. Despite the violation of homogeneity of covariance matrix and multivariate normality, the classification was sufficiently accurate to consider the discriminant analysis as satisfactory.

**Table 4:** Accuracy of prediction based on the discriminant functions.

	Classification results^a^			
	Predicted group membership			
Alliances (original count)	Cooperative	Collusive	Disordered	Total
Cooperative	21	1	0	22
Collusive	1	10	1	12
Disordered	0	2	8	10
% Cooperative	95.5	4.5	0	100
% Collusive	8.3	83.3	8.3	100
% Disordered	0	20	80	100

*^*a*^88.6% of original grouped cases correctly classified.*

Then, we resorted to a second statistical analysis to establish the internal consistency of the coding system FAAS-DCP. We conducted a principal components analysis (PCA) to determine the number of relevant components to evaluate family functioning. The Kaiser–Mayer–Olkin measure (KMO = 0.851) suggested that the correlation patterns were compact, and then the PCA should establish reliable and distinct factors. The Bartlett’s sphericity test indicated that if the matrix was not an identity matrix (χ^2^ = 259.86, df = 36, *p* < 0.05), then the dimensions correlated well with each other and they were not independent of each other. We carried out a PCA with an oblique rotation that highlighted two factors with an eigenvalue greater than 1 after extraction, explaining nearly 72% of total variance. The first factor accounted for 59% of the variance and the second factor for 13% of the variance. When the two factors were moderately correlated (*r* = 0.48), then we could rely upon the factors designed by the PCA. The first factor embraced the dimensions “readiness to interact,” “sensitivity,” “parental scaffolding,” “shared and co-constructed activities,” “gaze orientation,” “family warmth,” and “inclusion of partners.” It included physical adjustments, quality of stimulations addressed to the newborn, and the sharing of affect and activities. Moreover, the internal consistency was very good (*α* = 0.91), indicating that all dimensions contributed to the global measure of this factor. We named this factor “engagement and sharing.” The second factor embraced the dimensions “coparental coordination,” and “role organization,” also showing a very good internal consistency (*α* = 0.84). It included aspects related to how parents coordinate and support while adapting to the context. We named this factor “marital and coparental adjustment.”

### Study 3: Predictive Validity (*n* = 12)

The third study aimed at determining the existence of a link between triadic family functioning assessed at 3 weeks postpartum using the DCP, and the functioning assessed with the LTP at 3 months postpartum. Previous studies highlighted a stability of family functioning from pregnancy to the end of the 1st year postpartum (Favez et al., 2003, 2006a; Carneiro et al., 2006). Descriptive data highlighted that median scores were better in LTP than in DCP for mostly interactive dimensions (difference ranging from 1 to 3.5 points) except for the dimensions “parental scaffolding” and “sensitivity” on which the scores were better in DCP (differences ranging from 0.5 to 1.5 points). The non-parametric statistic of Wilcoxon signed-ranks was used and the effect size calculated to estimate the importance of the difference (see **Table 5**) as recommended by Field (2005).

**Table 5:** Wilcoxon signed-ranks test of shared interactive dimensions between FAAS-DCP and FAAS.

Dimensions DCP versus LTP	Positive ranks	Negative ranks	Ties	Z	Exact Sig. (2-tailed)	Effect size *r*
Readiness to interact	8	2	2	–1.740^a^	0.090	–0.35
Gaze orientation	7	4	1	–1.788^a^	0.080	–0.36
Inclusion of partners	4	7	1	–0.089^b^	0.948	–0.02
Coparental coordination	5	6	1	–0.090^b^	0.965	–0.02
Role organization	5	3	4	–0.775^a^	0.500	–0.16
Parental scaffolding	3	9	0	–2.139^b^	0.033	–0.44
Shared activities	4	7	1	0.000^c^	1.000	0.00
Sensitivity	5	5	2	–0.974^a^	0.385	–0.20
Family warmth	4	6	2	–0.157^b^	0.910	–0.03

*^*a*^Based on negative ranks; ^*b*^Based on positive ranks; ^*c*^The sum of negative ranks equals the sum of positive ranks.*

We noted that families showed mostly higher scores in the LTP than in the DCP without a statistically significant difference and a weak or moderate importance of this difference for the dimension “readiness to interact” (*Z* = -1.74, *p* > 0.05, *r* = -0.35), “gaze orientation” (*Z* = -1.79, *p* > .05, *r* = -0.36), “role organization” (*Z* = -0.77, *p* > .05, *r* = -0.16). The lack of difference and the weak or moderate effect size indicated that the improvement of scores was not relevant for these dimensions, which was in line of the stability of family functioning between 3 weeks and 3 months postpartum. Later, families showed mostly lower scores in the LTP than in the DCP without a statistically significant difference and an absence of importance in the difference for the dimensions “inclusion of partners” (*Z* = -0.08, *p* > 0.05, *r* = -0.02), “coparental coordination” (*Z* = -0.09, *p* > 0.05, *r* = -0.02), “shared and co-constructed activities” (*Z* = 0.00, *p* > 0.05, *r* = 0.00) and “family warmth” (*Z* = -0.16, *p* > 0.05, *r* = -0.03). We can conclude that the decrease in scores between the two observational situations was not relevant and hence, the triadic interactions assessed were similar at the two measurement points. Again, results argued for the stability of the triadic family functioning. The ratio of families showing an increase or a decrease in scores on the “sensitivity” dimension was balanced at both measurement points. The difference was not statistically significant and the effect size confirmed the lack of importance of this difference (*Z* = 0.00, *p* > 0.05, *r* = 0.00). Finally, “parental scaffolding” was the unique interactive dimension that presents a statistically significant difference, which was of moderate importance (*Z* = -2.14, *p* < 0.05, *r* = -0.44). The lack of stability was therefore acceptable.

Later, we examined the stability between DCP and LTP in the attribution of family alliances. Given the small size of our sample and in order to increase the statistical power, we combined the collusive and disordered alliances in one named “dysfunctional.” The cooperative alliance was named “functional.” **Table 6** shows that all families with a functional alliance in DCP had a functional alliance in LTP also. Most of the families with a dysfunctional alliance in DCP also had a dysfunctional alliance in LTP (83.3%). On the other hand, one family with a dysfunctional alliance in DCP showed an improvement in the family functioning, the alliance becoming functional in LTP (16.7%). **Table 6** also highlights that when our 4 cells have an expected count of less than 5, then we used the McNemar’s Chi-squared test as recommended by Brace et al. (2006). Results indicated an absence of difference for family alliances attributed in DCP and LTP [χ^2^(1) = 0, *p exact* > 0.05]. Consequently, family alliances were stable between 3 weeks and 3 months postpartum. Moreover, the association between the alliances classified in DCP and LTP was strong (Φ = 0.845), the family alliances in DCP explaining 71% of variance in the family alliances in LTP. In other words, these results highlight a strong stability in family alliances during the 1st months postpartum.

**Table 6:** Cross-tabulation between alliances recoded in two categories in DCP and LTP.

		Functional alliance in LTP	Dysfunctional alliance in LTP	Total
Functional alliance in DCP	Count	6	0	6
	Expected count	3.5	2.5	6
	% within the family alliance in DCP	100	0	100
Dysfunctional alliance in DCP	Count	1	5	6
	Expected count	3.5	2.5	6
	% within the family alliance in DCP	16.67	83.33	100
Total	Count	7	5	12
	Expected count	7	5	12
	% within the family alliance in DCP	58.33	41.67	100

## Discussion

Our intent was to present a new observational tool to assess early family interactions at a triadic level, including the mother, the father, and the newborn at an early age, with the aim of providing clinicians and researchers a way to identify potential family distress and offering an appropriate intervention to parents who need help in dealing with changes related to the transition to parenthood. Our three studies consisted to the first steps of validation with promising results.

A good inter-rater reliability was highlighted on each interactive dimension and for the alliance attribution as defined in the coding system FAAS-DCP. The dimensions “coparental coordination,” “role organization,” and “shared and co-constructed activities” could be improved with a better specification of behaviors taken into account in the assessment. It should be noted that these three dimensions are related to the construct of coparenting. Interferences and lack of coordination between parents, despite mutual support, may be more difficult to assess because their operationalisation is more adapted to older infants and parents that are more experienced in their roles. At 3 weeks postpartum, parents do not seem to be familiar yet with their new roles and they are still exploring their baby. Therefore, the interactions are less fluid and coordinated at 3 weeks than at 3 months. Also, the nature of the activity could elicit more interference behaviors because a parent could request the other parent’s help to change the diapers. Therefore, interference is not necessarily related to a lack of coordination or a lack of respect for the role, but could also be seen as a support provided between parents, and related to coparenting (McHale et al., 2002). This could explain some of the variations between raters.

Then, another element of validation was provided by the establishment of good reliability and internal consistency. We have confirmed that the structure of the coding system FAAS-DCP was consistent with the underlying theoretical construct. We have confirmed that the evaluation of family interactions correctly predicted the type of family alliance. The risk of making a mistake was acceptable, especially because risk was to confound with collusive and disordered alliances, that are both considered dysfunctional (Fivaz-Depeursinge et al., 1996; Frascarolo et al., 2005). Specifically, these categories of alliances are clinically related to problematic child’s development (Frascarolo et al., 2007b), which is why the misattribution between them is acceptable. Next, we have highlighted two factors with a good internal consistency that we designated, respectively, “engagement and sharing” and “marital and coparental adjustment.” The first factor embraces diverse aspects of family functioning reflecting the physical availability and inclusion of partners, intuitive parenting, and cognitive and affective exchanges. It is consistent with the idea that the parental frame includes varied, predictable and adapted stimulations, as well as regulation and validation of affects (Stern, 1999). In turn, this parental frame fosters participation by the newborn (Fivaz-Depeursinge and Corboz-Warnery, 2001) and the circulation of affects between family members. Consequently, this factor seems to be a measure of engagement of each family member and the quality of their exchanges. The involvement of parents with the newborn is one aspect of coparenting (McHale, 2007; Schoppe-Sullivan et al., 2016), and, in fact, is considered in the second factor. This divergence from the theoretical concept may be due to the point in time of the evaluation. The early interactions assessed at 3 weeks postpartum could reflect more parenting than coparenting behaviors.

Taken together, the two established factors and the inherent difficulties distinguishing what represents interference, lack of respect for a role, or mutual support (as showed with the inter-rater reliability), led us to reconsider the concept of coparenting in this specific period. We suggest considering coparenting as an “early coparenting” with a focus on support and confidences, rather than on coordination between parents. Additional research is necessary to understand this conceptualisation of coparenting and determine its legitimacy.

Our results are supported by the feedbacks of parents. Indeed, they have expressed to have much pleasure to do the DCP task. Specifically, they said that they rapidly forget the cameras and mostly had pleasure to spend some family time together. Nonetheless, they also expressed that it was difficult to respect the assigned roles during the diapering (parts 1 and 2). For example, some fathers said that they were eased to have help from their partner (e.g., the mother lays out the diaper while the father takes the clothes off). They perceive the mother’s intervention as a support. In the same way, some mothers perceived the father’s intervention as a sign of involvement. In fact, this kind of situations often occurred during the DCP. On the other hand, some fathers or others experienced the partner’s intervention as intrusive, even as a sign of lack of confidence. These examples stress the complexity to interpret behaviors as supportive or intrusive and then, to consider to respect or non-respect of roles. The fact that some parents openly expressed the need of intervention of the partner is consistent with the proposition to reconsider the concept of “early coparenting” as being mainly based on support and confidence, and less on coordination.

However, the highlighted second factor argues for the validity of this concept at early postpartum. In fact, the second factor embraces the coparental and marital functioning that are distinct but interrelated (McHale et al., 2002; Bonds and Gondoli, 2007; Delvecchio et al., 2015). As underlined by Fivaz-Depeursinge and Corboz-Warnery (2001), the conflict between parents is shown in the “coparental coordination” and in the “role organization,” the two dimensions underlying this factor. Consequently, this second factor is consistent with the theoretical conception of links between the coparental and marital subsystems. Moreover, taken together, the two factors allow a typology as follows: (1) high “engagement and sharing” and high “marital and coparental adjustment” describe families characterized by availability, inclusion of each member to allow affect sharing, and support between parents; (2) high “engagement and sharing” and low “marital and coparental adjustment” refers to families in which partners are sufficiently engaged in interactions, with adapted stimulation for a newborn to preserve the ability to validate newborn’s state, but marked by conflicting exchanges between parents that may lead to a coparental or even marital division; (3) low “engagement and sharing” and “marital and coparental adjustment” refers to families that could not engage in the interactions, which are characterized by exclusion of partners, a lack of support between parents, and a difficulty in adapt to the newborn’s state. This typology derived from our two factors is consistent with the typology of family alliances as described by Fivaz-Depeursinge and Corboz-Warnery (2001).

This typology was in accordance with our observations of interactive behaviors observed in the family system during the DCP. Despite the fact that families expressed pleasure to spend a triadic moment and the fact that they were videotaped, problematic and dysfunctional family functioning were easily identifiable. As it was showed in previous studies (Lindahl, 2001; Weiss and Heyman, 2004; Baker et al., 2010), some distressed parents could not fake that “everything is fine”, while it is not the case in everyday life. On a clinical level, it is interesting to note that observing families during the DCP allows an identification of those that are at risk to develop problematic or dysfunctional relationships within the family system. Our experience in observation of triadic interactions shows that it is crucial to consider affects, verbal and non-verbal cues to understand what happening within the family system. For example, when one parent intervene in the activity between the other parent and the newborn, if it is going along with positive shared affects, it will lead to positive exchanges between all members. On the other hand, when a similar intervention is going along with negative affects and withdrawal behaviors of one parent from the interaction, the continuation of triadic interactions is compromised. We could observe in this case conflicts between parents and/or exclusions behaviors. Negativity contaminated the exchanges between family members.

It is interesting to note that interactions coded with “FAAS – DCP” coding system were not related to pregnancy characteristics, such as pregnancy length or the kind of delivery. It seems that a preterm birth as well as cesarean versus vaginal delivery or any other obstetrical intervention like the use of ventouse, forceps or episiotomy doesn’t represent variables that could predict problematic and dysfunctional family functioning. This result is consistent with previous results that showed subjective childbirth experience being a better predictor of adaptative difficulties during the 1st months after delivery (Lemola et al., 2007; Gürber et al., 2012). Thus, the DCP and its coding system offer an original way to investigate some aspects of the early postpartum. It is a very useful tool, especially as medical variables related to pregnancy and delivery cannot explain the early triadic family functioning.

Given that we could detect distressed families through the observed triadic interactions during the DCP, the 1st month postpartum is a crucial period to intervene toward these families, especially as we had highlighted continuity in family functioning from this period to 3 months postpartum. Indeed, the predictive validity has shown that the family functioning is stable across time. Specifically, triadic interactions are similar at 3 weeks and at 3 months postpartum, as assessed with the common interactive dimensions of the two coding systems. This also applies for the establishment of family alliances. The coding system FAAS-DCP could be used to assess the triadic family functioning given the fact that our assessment fit into the continuity of the well-established stability from pregnancy to the end of the 1st year postpartum. Even if the differences are not significant, the improvement in interactive scores between DPC and LTP suggests that interactions are more fluid and coordinate as soon as the family experiences the transition to parenthood, which is again consistent with the idea of revisiting the coparenting definition in the early postpartum. Previous studies have described some families that experience negatively impacted family relationships, from pregnancy to the postnatal period, and rated them “high to low” (Favez et al., 2003). We have highlighted the existence of another group rated “low to high”. Taken together, these results focus on the disruptions occurring between the prenatal and postnatal period and the importance of specifically studying the quality of early interactions compared to the another assessment period.

Of note, feedbacks of parents could perhaps provide an explanation for our finding of a “low to high” group. Indeed, some parents reported that the diapering activity in the context of maternity ward help them to feel efficient in their parental role. Specifically, sharing this activity in a triadic context allows the parents to discuss together and to support each other, which fosters the sense of self-efficacy. Some fathers said that this situation allowed them to practice the caregiving task and share some interrogations with their partners, contributing in turn to feel confident and working in a secure context. In other words, the setting of DCP could allow triggering some resources within the family system promoting at the same time parental, coparental, and marital roles. It emphasizes the crucial role of communication between parents that are dealing with the transition to parenthood and the importance for them to find a way that allows supporting each other in their new roles. When the communicative skills are ineffective, we could observe this during the DCP and then, come back to it with parents through discussion and feed-back. Hence, the setting of DCP provide the opportunity for both clinicians and researchers to promote an early identification of family distress and to offer new therapeutic settings, like video feedback, to discuss of difficulties and resources within the family system (Frascarolo et al., 2009, 2011; Favez et al., 2012a) during the limited time when perinatal caregivers are in contact with families (Ayers et al., 2007; Rime and Stadlmayr, 2013). The fact that diapering corresponds to a daily activity was experienced by parents as a relevant setting allowing to engage discussion both between them and with professional caregivers. According to their experience during the DCP, this observational situation allows parents to talk about how they experience the transition to parenthood while working on diapering in order to foster the sense of parental self-efficacy. These first steps toward the validation of the DCP are promising, especially as the caregiving task could be a good way to detect potentially distressed families as well as a gateway to offer them some practical support. Future studies using the DCP might consider to extend its external validity by linking this observational assessment of the family alliance to other self-reported measures of parental functioning. Indeed, variables such as parental personality or perceived stress, might allow to explain the differences in family functioning observed in the DCP. Convergent results between observational and self-reported data would bring an even stronger evidence toward the validity of the DCP.

## Author Contributions

JR made substantial contributions to the conception of the work, the analysis and interpretation of data, and drafting the work, gave the final approval of the version to be published, and agreed to be accountable for all aspects of the work in ensuring that questions related to the accuracy or integrity of any part of the work are appropriately investigated and resolved. HT revised the work critically for important intellectual content. NF made substantial contributions to the conception of the work and to revising the work critically for important intellectual content. MW revised the work critically for important intellectual content. WS made substantial contributions to the conception of the work, revised the work critically for important intellectual content, and gave the final approval for the version to be published.

## Conflict of Interest Statement

The authors declare that the research was conducted in the absence of any commercial or financial relationships that could be construed as a potential conflict of interest.

## Footnotes

1 The cooperative alliance could be subdivided in two categories. When parents are well-coordinated and interactions are fluids, the cooperative alliance is called “harmonious.” When some mismatches during interactions are punctually observed (for example at body orientation level, or when one or both parents over or under-stimulate the infant), and interactions are less fluids (i.e., co-constructed activities are realized or maintained with difficulties), the cooperative alliance is called “stressed.” In our study, we did not distinguish between these two sub-categories to increase the statistical power.
